# Neurophysiological measures of nociceptive brain activity in the newborn infant – the next steps

**DOI:** 10.1111/apa.12490

**Published:** 2014-02-04

**Authors:** Caroline Hartley, Rebeccah Slater

**Affiliations:** Department of Paediatrics, University of OxfordOxford, UK

**Keywords:** Brain, Infant, Nociception, Pain

## Abstract

Infants within neonatal intensive care units can receive multiple medically essential painful procedures per day. How they respond to these events, how best to alleviate the negative effects, and the long-term consequences for the infant are all significant questions that have yet to be fully answered. In recent years, several studies have examined cortical responses to noxious stimuli in the neonate through the use of near-infrared spectroscopy (NIRS) and electroencephalography (EEG). These investigations dispel any notion that the newborn infant does not process noxious stimuli at a cortical level and open the way for future research. In this Viewpoint Article, we review these studies and discuss key clinical challenges which may be elucidated with the use of these techniques.

**Conclusion:**

Simultaneously measuring the changes that are evoked in behaviour, physiology and the cortex following noxious events will provide the best approach to understanding the neonate's experience of pain.

Pain is an emotional subjective response to a (potential or actual) tissue damaging procedure (1). As such, it is not possible to determine directly whether non-verbal patients are in pain, or whether they find certain stimuli more painful than others. This is a particular issue in the neonatal population where the nervous system is developing (2) and responses to nociceptive stimuli are known to differ from those observed in the adult (3). While scepticism towards the existence of infant pain, which dominated 20th century literature, is no longer a prevailing view, researchers and clinicians still struggle to measure pain effectively in infants (4). A large number of studies aim to quantify infants' responses to noxious procedures and aid clinicians in the treatment of pain [for reviews, see Ranger et al. (5) and Anand et al. (6)]. In this Viewpoint Article, we will discuss the notion that human infant pain may be best understood by combining measures of neonatal brain activity with other well-characterised behavioural and physiological indicators of pain. This approach may provide the best composite measure of an infant's pain experience.

In the past decade, several studies have used neurophysiological techniques to measure cortical responses to noxious stimuli. Studies using near-infrared spectroscopy (NIRS) have shown increases in haemoglobin concentration over central regions following acute noxious stimuli (7,8). Electroencephalography (EEG) recordings have demonstrated an increase in power in right frontal regions (9) and evoked responses over central regions (10,11). This evoked activity is noxious-specific in term infants (10), while non-specific delta brush activity is predominantly observed in response to both noxious and tactile stimulation before 35-weeks gestation (11). Cortical activation is a fundamental component of pain processing widely demonstrated in the adult literature (12). Thus, the observation that the youngest infants are able to manifest cortical responses to noxious stimuli is of clear importance and challenges any notion of the decorticate neonate, proposed by Sherman et al. in 1936 (13) and still common in the late 1960s (14).

Additionally, as cortical processing is an important component of conscious awareness, it has been suggested that these observations give credence to consciousness in the newborn (15) and may reflect pain perception. However, to date, this work provides only the first steps in understanding how human neonates process noxious stimuli at a cortical level. The advantages and disadvantages of these techniques will be discussed in terms of their relevance to the study of neonatal pain and future directions are considered.

## A COMPOSITE MEASURE OF NEONATAL PAIN

Electroencephalography and NIRS are non-invasive techniques that can be used to monitor brain activity from short periods up to several days. Recordings can be carried out alongside clinical care with portable recording equipment, and for this reason, they are ideally suited to the environment of neonatal wards. Both techniques, however, require trained staff to conduct and interpret the recordings. Behavioural and physiological measures (5) are more easily and efficiently obtained and in this respect are advantageous to EEG and NIRS for clinical assessment of pain. So what can be gained in the clinical setting from neurophysiological studies? As with any complex process, our best understanding of infant pain can be achieved through the assessment and integration of multiple measures. However, as cortical processing is required for the perception of pain, recording cortical activity evoked by noxious events is perhaps the closest we can get to a physiological measure of pain. It is therefore beneficial that research studies are undertaken that aim to identify the physiological and behavioural measures that best correlate with nociceptive-specific changes in cortical brain activity. Indeed, while many studies have examined behavioural and physiological responses to noxious procedures, there is limited consensus within the literature as to the most appropriate measure (or measures) for quantifying neonatal pain (5). Measuring cortical activity concurrently with behavioural and physiological measures may help identify the best clinically practical measures of infant pain (16).

**Figure 1:**
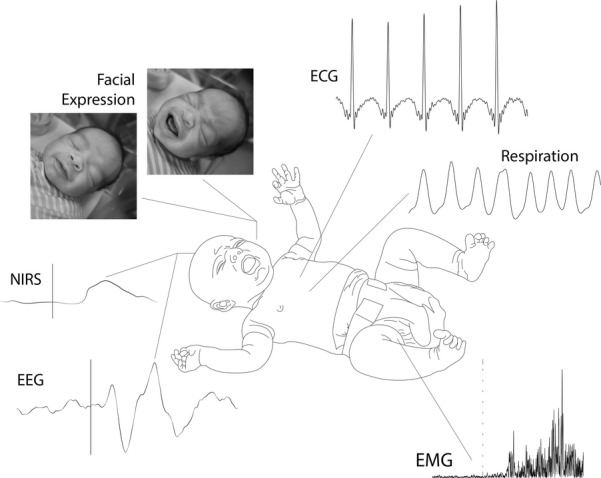
Schematic of the range of recording measures [electroencephalography (EEG), near-infrared spectroscopy (NIRS), EMG, ECG, respiration, change in facial expression] that can be used to quantify nociceptive processing in the infant nervous system.

Good agreement has been shown between premature infant pain profile (PIPP) scores, in particular facial expression, and cortical activity recorded using NIRS (17). However, it is important to note that some infants without a change in facial expression still demonstrated a localised change in haemoglobin concentration in the contralateral somatosensory cortex (17). This result highlights the advantages of analysing cortical activity compared with behavioural measures and the additional information that such analysis may yield. Correlations between evoked activity recorded using EEG and facial expression or other measures of behaviour have not been investigated [other than with sucrose administration (18)]. We propose that future research studies should continue to examine behavioural indicators of pain – the way the infant interacts with their environment and communicates their experience with others is of clear importance – but that these measures should be combined with the analysis of nociceptive-specific brain and spinal cord activity (Fig.  1). While all these measures are necessarily surrogate indicators of pain, which by its nature is a subjective experience modulated by environmental and psychological factors (12), understanding the way that nociceptive inputs are processed at all levels of the nervous system and how this experience is manifest behaviourally will improve the treatment and understanding of infant pain. Thus, while it is neither feasible nor necessary to assess cortical or spinally mediated activity in all infants, we suggest that current research efforts should focus in this area to maximise the clinical potential that may come from these investigations. Methods for simultaneous recordings of neurophysiological, behavioural and physiological responses are described by Worley, Fabrizi and colleagues (19,20).

## CONSEQUENCES OF NEONATAL PAIN

Early-life pain may have long-term consequences on subsequent pain experience, with altered responses to nociceptive stimuli reported later in life (21–23). Relatively short-term alterations in nociceptive processing have also been identified using EEG: subjects born prematurely (who will have been subject to a number of medically essential noxious stimuli during the premature period) have an increased evoked response at term-corrected age compared with term-born controls (24). However, it is not known whether this change is related to the number of previous nociceptive events, or whether it is a direct consequence of receiving noxious procedures during a particular developmental period. Furthermore, children born very prematurely are more likely to have cognitive difficulties (25). It is plausible that these problems relate to abnormal exposure to stimuli in the *ex utero* environment at a critical phase of development. While this is speculative and the mechanisms that underlie these observations are not fully understood, there are some indications that pain experienced in early life may have adverse outcomes, such as poorer cognition or alterations in sensory processing. Indeed, neuroimaging techniques have begun to show evidence that corroborates this. Neonatal skin breaking (26) and stressful (27) procedures are associated with abnormal brain development within the neonatal period. Recently, Doesburg et al. (28) demonstrated a link between neonatal skin breaking procedures, functional brain activity and visual perceptual ability in school-age children born at extremely low gestational age. They found no association with older preterm and term-born children suggesting an early period of heightened vulnerability to neonatal pain-related stress. A key question is whether direct measurements of nociceptive brain activity in the neonatal period can improve our understanding of how these abnormal outcomes arise.

Given the possible long-term consequences of neonatal pain, and the short-term distress associated with these procedures, it is important that we provide pain relief during noxious events. While studies have shown changes in behavioural and physiological measures in relation to pain management techniques, it is important to remember that these changes may not be correlated with the underlying nociceptive activity in the brain and spinal cord. Indeed, there was no difference in the cortical evoked response in a randomised controlled trial of neonates receiving oral sucrose compared with those who received sterile water (18). This was despite the usual reduction in facial expression scores (18,29) in the group who received sucrose. Methods of pain relief that result in a diminished behavioural response may imply that there is an altered experience of the noxious event; however, if the nociceptive input is still reaching the brain, then negative short-term effects and long-lasting consequences may still prevail. Indeed, Taddio and colleagues demonstrated that hyperalgesia caused by repeated blood tests performed in the first 2 days of life was not alleviated by the administration of sucrose (30). Conversely, if a particular analgesic is found to alter cortical activity but does not change the observed physiological or behavioural measures (compared with controls), then the reflexive and/or autonomic responses, which can have important health consequences for the infant, still need to be addressed.

## CHARACTERISING NOCICEPTION IN THE CLINICAL SETTING

The sickest infants may be unable to mount a behavioural response to a painful procedure (due to medication, obstructive procedures such as ventilators or lack of energy). They may also have neurological complications leading to altered neuronal responses to noxious stimuli. In postasphyxic and very preterm infants, somatosensory evoked potentials have been shown to have prognostic value for neurological sequelae and future cerebral palsy (31,32). It has therefore been suggested that EEG and evoked potentials be used routinely as a clinical assessment tool in the neonatal period (32). However, neonates with specific pathologies have yet to be examined using neurophysiological techniques in relation to noxious processing. This is important for the treatment of these infants, who are likely to require the largest number of noxious procedures during their time in neonatal intensive care.

The neurophysiological studies to date have in the most part focused on acute noxious stimuli (heel lances and venopuncture). However, a study conducted by Limperopoulos et al. (33) showed an increased haemodynamic response to a variety of clinically required procedures, with the greatest response during endotracheal tube repositioning and ‘complex caregiving events’. Due to the prolonged nature of some procedures, it is possible that they present more of a ‘risk’ to the developing infant brain than many of the acute procedures that have been most well studied. Future investigations examining longer clinical procedures would be of great benefit to our understanding of the cortical processing of noxious stimuli. Moreover, prolonged pain, for example, postoperative pain, also presents a problem on the neonatal ward. Future work should examine whether prolonged pain alters the response to acute noxious stimuli, as well as the neurological signatures associated with prolonged pain. EEG and NIRS provide objective quantitative approaches for investigating these questions. When combined with simultaneous recordings of behavioural and physiological measures, such research would lead to a better understanding of ongoing pain in neonates.

## CONCLUSION

Electroencephalography and NIRS provide a non-invasive ‘window into the brain’, of key importance for understanding the rapidly developing human infant nervous system. These techniques can be used to examine cortical activity in response to noxious stimuli, and we suggest that future research examining the processing of noxious stimuli in infants incorporate these measures in combination with physiological and behavioural indicators of pain. Simultaneous recordings of multiple measures will provide a more complete picture of the response to a procedure and how this response may be affected by analgesics. Future work should also investigate prolonged painful experiences, noxious processing in pathological states and the network of brain regions involved in processing noxious stimuli at different developmental stages. The studies conducted thus far provide a significant starting point in our understanding of cortical pain processing in the newborn infant. As quantitative measures of cortical activity are perhaps the closest we can get to understanding pain in non-verbal populations, these techniques have strong potential for future research. While it may be unfeasible to use these techniques directly within clinical protocols, the studies we have suggested here should lead to improved pain management strategies for newborn infants.

## References

[1] Merskey H, Bogduk N (2011). Classification of chronic pain. Task force on Taxonomy.

[2] Kostovic I, Rakic P (1990). Developmental history of the transient subplate zone in the visual and somatosensory cortex of the macaque monkey and human brain. J Comp Neurol.

[3] Fitzgerald M (2005). The development of nociceptive circuits. Nat Rev Neurosci.

[4] Rodkey EN, Pillai Riddell R (2013). The infancy of infant pain research: the experimental origins of infant pain denial. J Pain.

[5] Ranger M, Johnston CC, Anand KJS (2007). Current controversies regarding pain assessment in neonates. Semin Perinatol.

[6] Anand KJS, Stevens BJ, McGrath PJ (2007). Pain in neonates and infants.

[7] Bartocci M, Bergqvist LL, Lagercrantz H, Anand KJS (2006). Pain activates cortical areas in the preterm newborn brain. Pain.

[8] Slater R, Cantarella A, Gallella S, Worley A, Boyd S, Meek J (2006). Cortical pain responses in human infants. J Neurosci.

[9] Fernandez M, Blass EM, Hernandez-Reif M, Field T, Diego M, Sanders C (2003). Sucrose attenuates a negative electroencephalographic response to an aversive stimulus for newborns. J Dev Behav Pediatr.

[10] Slater R, Worley A, Fabrizi L, Roberts S, Meek J, Boyd S (2010). Evoked potentials generated by noxious stimulation in the human infant brain. Eur J Pain.

[11] Fabrizi L, Slater R, Worley A, Meek J, Boyd S, Olhede S (2011). A shift in sensory processing that enables the developing human brain to discriminate touch from pain. Curr Biol.

[12] Tracey I, Mantyh PW (2007). The cerebral signature for pain perception and its modulation. Neuron.

[13] Sherman M, Sherman L, Flory CD (1936). Infant behavior. Comp Psychol Monogr.

[14] Swafford LL, Allan D (1968). Pain relief in the pediatric patient. Med Clin North Am.

[15] Lagercrantz H, Changeux J-P (2010). Basic consciousness of the newborn. Semin Perinatol.

[16] Holsti L, Grunau RE, Shany E (2011). Assessing pain in preterm infants in the neonatal intensive care unit: moving to a ‘brain-oriented’ approach. Pain Manag.

[17] Slater R, Cantarella A, Franck L, Meek J, Fitzgerald M (2008). How well do clinical pain assessment tools reflect pain in infants?. PLoS Med.

[18] Slater R, Cornelissen L, Fabrizi L, Patten D, Yoxen J, Worley A (2010). Oral sucrose as an analgesic drug for procedural pain in newborn infants: a randomised controlled trial. Lancet.

[19] Worley A, Fabrizi L, Boyd S, Slater R (2012). Multi-modal pain measurements in infants. J Neurosci Methods.

[20] Fabrizi L, Worley A, Patten D, Holdridge S, Cornelissen L, Meek J (2011). Electrophysiological measurements and analysis of nociception in human infants. J Vis Exp.

[21] Taddio A, Katz J, Ilersich AL, Koren G (1997). Effect of neonatal circumcision on pain response during subsequent routine vaccination. Lancet.

[22] Fitzgerald M, Walker SM (2009). Infant pain management: a developmental neurobiological approach. Nat Clin Pract Neurol.

[23] Hermann C, Hohmeister J, Demirakca S, Zohsel K, Flor H (2006). Long-term alteration of pain sensitivity in school-aged children with early pain experiences. Pain.

[24] Slater R, Fabrizi L, Worley A, Meek J, Boyd S, Fitzgerald M (2010). Premature infants display increased noxious-evoked neuronal activity in the brain compared to healthy age-matched term-born infants. Neuroimage.

[25] Allen MC (2008). Neurodevelopmental outcomes of preterm infants. Curr Opin Neurol.

[26] Brummelte S, Grunau RE, Chau V, Poskitt KJ, Brant R, Vinall J (2012). Procedural pain and brain development in premature newborns. Ann Neurol.

[27] Smith GC, Gutovich J, Smyser C, Pineda R, Newham C, Tjoeng TH (2011). Neonatal intensive care unit stress is associated with brain development in preterm infants. Ann Neurol.

[28] Doesburg SM, Chau CM, Cheung TPL, Moiseev A, Ribary U, Herdman AT (2013). Neonatal pain-related stress, functional cortical activity and visual-perceptual abilities in school-age children born at extremely low gestational age. Pain.

[29] Stevens B, Yamada J, Lee GY, Ohlsson A (2013). Sucrose for analgesia in newborn infants undergoing painful procedures. Cochrane Database of Syst Rev.

[30] Taddio A, Shah V, Atenafu E, Katz J (2009). Influence of repeated painful procedures and sucrose analgesia on the development of hyperalgesia in newborn infants. Pain.

[31] Majnemer A, Rosenblatt B, Riley PS (1990). Prognostic significance of multimodality evoked response testing in high-risk newborns. Pediatr Neurol.

[32] Vanhatalo S, Lauronen L (2006). Neonatal SEP – back to bedside with basic science. Semin Fetal Neonatal Med.

[33] Limperopoulos C, Gauvreau KK, O'Leary H, Moore M, Bassan H, Eichenwald EC (2008). Cerebral hemodynamic changes during intensive care of preterm infants. Pediatrics.

